# High prevalence of ACE DD genotype among north Indian end stage renal disease patients

**DOI:** 10.1186/1471-2369-7-15

**Published:** 2006-10-17

**Authors:** Gaurav Tripathi, Poonam Dharmani, Faisal Khan, RK Sharma, Vinod Pandirikkal Baburajan, Suraksha Agrawal

**Affiliations:** 1 Department of Medical Genetics, Department of Medical Genetics, Sanjay Gandhi Post Graduate Institute of Medical Sciences, Lucknow (UP) 226014, India; 2 Department of Nephrology, Sanjay Gandhi Post Graduate Institute of Medical Sciences, Lucknow (UP) 226014, India

## Abstract

**Background:**

The Renin-Angiotensin system (RAS) is a key regulator of both blood pressure and kidney functions and their interaction. In such a situation, genetic variability in the genes of different components of RAS is likely to contribute for its heterogeneous association in the renal disease patients. Angiotensin converting enzyme-1 (ACE-1) is an important component of RAS which determines the vasoactive peptide Angiotensin-II.

**Methods:**

In the present study, we have investigated 127 ESRD patients and 150 normal healthy controls from north India to deduce the association between ACE gene polymorphism and ESRD. The inclusion criteria for patients included a constantly elevated serum creatinine level above normal range (ranging from 3.4 to 15.8) and further the patients were recommended for renal transplantation. A total of 150 normal healthy controls were also genotyped for ACE I/D polymorphism. The criterion of defining control sample as normal was totally based on the absence of any kidney disease determined from the serum creatinin level. Genotyping of ACE I/D were assayed by polymerase chain reaction (PCR) based DNA amplification using specific flanking primers Based on the method described elsewhere.

**Results:**

The difference of DD and II genotypes was found highly significant among the two groups (p = 0.025; OR = 3.524; 95%CI = 1.54-8.07). The combined genotype DD v/s ID+II comparison validated that DD genotype is a high risk genotype for ESRD (p = 0.001; OR = 5.74; 95%CI limit = 3.4-8.5). However, no correlation was obtained for different biochemical parameters of lipid profile and renal function among DD and non DD genotype. Interestingly, ~87% of the DD ESRD patients were found hypertensive in comparison to the 65% patients of non DD genotype

**Conclusion:**

Based on these observations we conclude that ACE DD genotype implicate a strong possible role in the hypertensive state and in renal damage among north Indians. The study will help in predetermining the timing, type and doses of anti-hypertensive therapy for ESRD patients.

## Background

End stage renal disease (ESRD) is a complex disorder encompassing a large variety of phenotypes. Each phenotype is a result of an underline kidney disease and superimposing environmental and genetic factors. The complexity of the phenotypic makeup of renal diseases makes it difficult to diagnose and predict their progression and to decide on the optimal treatment for each patient. ESRD is an advanced form of chronic renal failure where renal function has declined to approximately 10% of normal prior to initiation of dialysis or transplantation. The impact of genetic variability on the development of renal failure is becoming clearer and emphasizes the need to elucidate the genetic basis for renal diseases and its complications. This would lead to the better understanding of different phenotypes observed in ESRD and would enable us to determine whether a patient is genetically predisposed to such complications.

Renal functions and blood pressure are tightly linked. Physiologically, kidneys provide a key mechanism of chronic blood pressure control via their infinite gain mechanism [1], whereas elevated blood pressure affects renal function via pressure natriuresis mechanism [2,3]. Pathophysiologically, long standing hypertension attenuates pressure natriuresis [4] and can cause or at least contribute to renal damage [5]. Therefore, hypertension is one of the imperative contributing factors associated with both causation and progression of renal failure [6]. It is a common, polygenic and complex disorder resulting from interaction of several genes with each other and with environmental factors [7].

The Renin-Angiotensin system (RAS) is a key regulator of both blood pressure and kidney functions and may play a role in their interaction. Its role in the pathogenesis of hypertension is well documented but its contribution to chronic renal failure and progression of kidney nephropathy is still debated [8]. It has been seen that RAS blockers i.e. both angiotensin converting enzyme (ACE) inhibitors and angiotensin receptor blockers lower blood pressure and can also attenuate or prevent renal damage [9]. However, major inter individual treatment responses to RAS inhibitors have been noted [10] and it remains difficult to predict responders based on known pathophysiological characteristics [11]. In such a situation, genetic variability in the genes of different components of RAS is likely to contribute for its heterogeneous association in the renal disease patients.

Angiotensin converting enzyme-1 (ACE-1) is an important component of RAS and it determines the vasoactive peptide Angiotensin-II. Its inhibition reduces the pace of progression of majority of chronic nephropathies [12,13]. The gene coding for ACE is subjected to an insertion/deletion (I/D) polymorphism that is a main determinant of plasma and tissue ACE levels [14]. Presence (insertion-I) or absence (deletion -D) of a 287 bp fragment in the 16^th ^intron of ACE gene has been linked to high prevalence of renal disorders among hypertensives and has been studied extensively [15]. Furthermore, the D allele has been linked to a failure of the renoprotective action of ACE inhibitors to retard the development of end stage renal disease (ESRD) [16,17]. Despite of the fact that most of the recent studies have suggested high prevalence of D allele among hypertensive individuals [13,18], still there are contradictory reports available [19]. This inconsistency could be in part due to the genetic and environmental heterogeneity among different ethnic groups [20].

In the present study, we have investigated the association between ACE gene polymorphism and the causation of renal disease in 127 end stage renal disease patients from north India. The major aim of the study was to explore whether the limited observations of association of ACE genotypes and renal function in patients of different ethnicities can be extended to all patients with primary renal disease among North Indians.

## Methods

### Subjects

Patients included in the present study were selected from the Department of Nephrology, which is one of the super specialty centres in Sanjay Gandhi Post Graduate Institute of Medical Sciences (SGPGIMS), Lucknow. The inclusion criteria for patients included a constantly elevated serum creatinine level above normal range (ranging from 3.4 to 15.8) and further the patients were recommended for renal transplantation. For each of the patient, the information was collected for various other criterion too that included age, gender, protein urea level, systolic and diastolic blood pressure and complete lipid profile (cholesterol (TC), triglycerides (TG), HDL, LDL and VLDL). Depending on the type and the severity of renal disorders, patients were categories into chronic glomerular nephropathy (CGN; n = 76), chronic intestinal nephropathy (CIN; n = 31), Hypertensive nephrosclerosis (HN; n = 2) and polycystic kidney (PK; n = 3). A total number of 127 patients were included in the study. All patients with Diabetic nephropathy were excluded from the study. A total of 150 normal healthy controls were also genotyped for ACE I/D polymorphism. A written consent was obtained from the patients and the controls and it was documented in the detailed performa. The controls were age, sex and ethnically matched. The study was approved by the Ethical committee of SGPGIMS and department of biotechnology, government of India.

### Blood collection

Blood samples for measuring Serum biochemical and lipid profiles were obtained in the morning. Patients were fasted for 8 hours. 3 ml of venous blood sample was collected in EDTA vials for the extraction of genomic DNA.

### DNA extraction

DNA was extracted from blood by salting out method using phenol-chloroform as described by **Coomey et al, 1994 **[21] and was purified by ethanol precipitation. DNA was used as a template for ACE polymorphism analysis.

### Determination of ACE genotyping

Genotyping of ACE I/D were assayed by polymerase chain reaction (PCR) based DNA amplification using specific flanking primers described elsewhere [8]. The primer sequences were as follows: Sense primer: 5' CTGGAGACCACTCCCAT CCTTTCT 3' and antisense primer: 5' GATGTGGCCATCACATTCGTCAGAT 3'. PCR reaction was performed in a final volume of 15 μl containing 5 pM/sample of primers, 0.25 mM/sample dNTPs, DNA buffer 1X/sample, 1U/sample Taq polymerase and 50 ng of genomic DNA. The DNA was amplified for initial denaturation at 94°C for 5 min followed by 35 cycles of 94°C for 1 min, 58.5°C for 90 min and 65°C for 4 min following a final extension of 72°C for 7 min (PTC 100, M J Research, Peltier thermal cycler). In order to avoid the mistyping of ID genotype as DD due preferential amplification of the shorter D allele, a separate PCR was carried out in all the DD samples.

**Figure 1:**
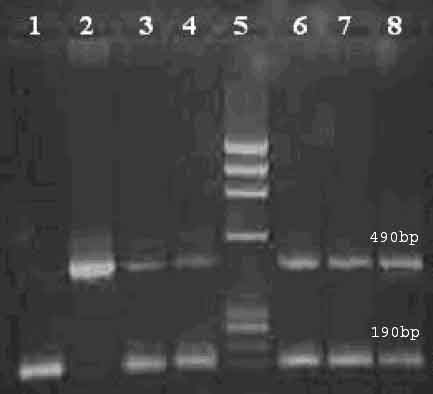
**Figure illustrating homozygous DD, homozygous II and heterozygous ID genotype**. Lane1: Homozygous DD sample. Lane 2: Homozygous II sample. Lane 3–4, 6–8: Heterozygous ID samples. Lane 5: DNA ladder (φ HindIII digest)

The PCR amplicon were genotyped by separating them on 2 % agarose gel electrophoresis and visualizing with ethidium bromide staining. The products were of the size 190 bp and 490 bp for I and D allele respectively. Hence, single bands of 190 and 490 bp confirmed homozygous DD and II genotypic state whereas two bands of 190 and 490 bp confirmed heterozygous ID genotype [Figure 1]. The allele sizing was carried out by using φHindIII digest DNA ladder (Amersham Biosciences).

### Statistical analysis

All the statistical calculation for the continuous data of biochemical and physiological factors were performed using SPSS version 10 statistical software packages. For each variable, the values are expressed as mean ± S D. Data was evaluated by One-Way Analysis of Variance (ANOVA) followed by Tukey's multiple comparison test. Allele and genotypic frequencies for ACE I and D alleles were calculated with the gene counting method. Comparison of the categorical data i.e. different ACE genotypes among controls and patients was done by Fischer's exact test and χ^2 ^test. Odd's ratios were calculated with a 95% confidence interval limit from 2 × 2 contingency table. ''*P*'' value < 0.05 was considered significant.

## Results

### Distributions of ACE genotypes

The distribution of DD, II and ID genotypes in the control group (n = 150) was 10 (6.7%), 100 (66.7%) and 40(26.6%) respectively, whereas among patient group (n = 127), 37 DD (29.13%), 48 II (37.79%) and 42 ID (33.07%) patients were observed [Table 1] The difference of DD and II genotypes was highly significant among the two groups (p = 0.025). This clearly established that patients with DD genotype are at high risk of developing renal disease (OR = 3.524; 95%CI = 1.54-8.07). Further, we have analyzed the data by pooling the ID genotype with II and DD genotypes respectively. It was observed that DD v/s ID+II comparison among the two groups were significantly different (p = 0.0001) and clearly ascertain that DD genotype is a high risk genotype as the OR value was also found to be as high as 5.74 (95%CI limit = 3.4-8.5). Even when the heterozygous ID genotype was pooled with DD then also the OR remained very high (OR = 3.826; 95%CI = 2.04-7.15).

**Table 1:** Distribution of ACE I/D genotypes among ESRD patients and controls

	**Patient (n = 127)**	**Control (n = 150)**
**Genotypes**		
**DD**	37(29.13%)	10 (6.7%)
**II**	48(37.79%)	100 (66.7%)
**ID**	42 (33.07%)	40 (26.6%)
**Alelles**		
**I**	138	240
**D**	116	60
**Comparison of different allelic and genotypic states**		
	**p- value**	**OR (95% CI limit)**
**D v/s I**	p = 0.0001	3.362 (2.3–4.8)
**DD v/s II**	p = 0.025	3.524 (1.54–8.07)
**DD v/s ID+II**	p = 0.0001	5.74 (3.4–8.5)
**DD+ID v/s II**	p = 0.0001	3.826 (2.04–7.15)

These highly significant differences observed among control and patient groups at the genotypic level were also visible at the allelic level [Table 1] as D allele was found in a frequency of 0.2 among controls and was more than double among patients as its frequency was found to be 0.45 (p = 0.0001; OR = 3.362; 95%CI = 2.3-4.8).

### Clinical characteristics of ESRD patients with different ACE genotypes

In order to asses the cumulative affect of ACE gene polymorphism with other risk factors; we compared various clinical parameters of the ESRD patients among two genotypic groups, DD and ID+II [Table 2]. It was observed that neither mean age nor any of the five lipid parameters namely TC, TG, HDL, LDL and VLDL differs significantly among the two sub-groups (p > 0.05). Overall, the mean age of the patients was found to be 35.32 ± 9.96. Similarly the mean value of TC, TG, HDL, LDL and VLDL was found to be 161.65 ± 3.3, 150.84 ± 5.7, 39.92 ± 0.84, 92.427 ± 2.61 and 34.07 ± 1.5 respectively [Table 2]. Similarly, when two important renal function parameters i.e. serum creatinin levels and protein urea were compared among the two sub groups, the differences were found to be non-significant. The mean value of the serum creatinin and protein urea among ESRD patients was found to be 8.38+ 0.25 and 2.76 + 0.09 respectively [Table 2]. However, when we compared the number of hypertensive patients among the two sub groups it was noticeably evident that ~87% of the DD genotype patients were hypertensive as compared to the 65% of II+ID genotype group (P = 0.026). The results further confirmed the association of DD genotype with the hypertensive state and implicate a strong possible role in renal damage.

**Table 2:** Clinical characteristics of ESRD patients with different ACE genotypes

	**Total patients**	**DD (n = 37)**	**II+ID (n = 90)**	**p-value**
**Mean Age**	35.32 +9.96	35+1.7	35.45+0.99	ns
**Lipid profile**				
**TC**	161.65+3.350	163.74+5.62	160.37 + 4.273	ns
**TG**	150.84+ 5.715	159.12+ 11.48	151.52 + 6.72	ns
**HDL**	39.92+0.8484	36.71+ 1.038	37.64 + 0.9716	ns
**LDL**	92.427+ 2.619	96.17+ 4.792	91.61 + 3.044	ns
**VLDL**	34.07+ 1.519	38.41+ 3.185	32.22+ 1.237	ns
**Serum Creatinine**	8.38+ 0.2553	8.43 + 0.49	8.41 + 0.2965	ns
**Protein Urea**	2.768 + 0.09	2.68 + 0.1969	2.97 + 0.106	ns
**Hypertensive %**	75.12%	86.48%	65.55%	0.0264

## Discussion

The data presented in this study is the first report from north India regarding the role of genetic variants of ACE gene in causation and progression of renal diseases. The findings clearly establish the association of ACE I/D gene polymorphism with the renal failure. The DD genotype was found to be a major risk determinants of ESRD among north Indians (OR = 5.74). Simultaneously, it was also observed that the hypertensive state is an important physiological state that affects the causation or progression of renal diseases.

Hypertension being a complex polygenic disorder is often regarded as a physiological state affected by "Genetic Predisposition", which highlights the presence of heritable allelic differences in the genes coding/associated with different components of RAS. Such differences result into differential transcript and protein expression accounting for different rates of progression of hypertension and other related diseases mainly, renal failures [22]. Among different RAS genes like angiotensin (AGT), angiotensin II type-1 receptor (AGTR1), rennin and ACE, the I/D polymorphism of ACE has been reported as a crucial determinant. The DD genotype have unanimously been shown to have increased serum ACE production and activity while II and ID genotypes produces low and intermediate levels of proteins respectively [22].

Angiotensin II has a potentially important role in the development of Glomerusclerosis [23] through its action as a growth factor and regulator of the cell growth and matrix production [24,25]. It has also been implicated that the inhibition of its production attenuates the progression of diabetic and non -diabetic nephropathies [26,27]. In this regard the importance of ACE and its genetic variants becomes more apparent. Although most of the studies on ACE I/D polymorphism have been very encouraging with regard to the role of DD genotype in the pathophysiology and treatment of diabetic nephropathies [28]. Similar studies in other types of nephropathies have yielded inconsistent results. For examples studies on autosomal dominant polycystic kidney patients have reported adverse effects of the D allele of the ACE gene in some cases [29,30], whereas number of other studies did not confirmed such association [31,32]. Similarly, an adverse effect of D allele was also found in some studies in IgA nephropathy or ESRD in general [33]. In a study of 80 family trios (proband and parents) with interstitial nephritis, the D allele was transmitted significantly and more frequently than would have been expected if no association existed. Further more, the ID and the DD genotypes were associated with a faster rate of renal function decline [34].

Our study revealed a highly significant difference in the presence of DD genotype and D allele of ACE gene among ESRD patients and normal controls validating that the ACE gene polymorphism is an important genetic determinant of non-diabetic nephropathies too. Overall findings were demarcating that D allele of ACE gene confers a high risk of developing renal diseases (OR = 3.36) and this association was highly compounded when D allele was present in homozygous state (OR = 5.74). Even inclusion of the heterozygous ID state known to have intermediate levels of ACE production along with the DD genotype depicted a high risk of renal failures (OR = 3.8). Therefore the finding that ACE DD genotype and D allele is associated with renal ESRD is likely to be true for the north Indian populations.

Furthermore, we postulate that DD genotype confers a greater role in hypertensive state as ~87% of DD genotype patients were hypertensive and this phenomenon could might have been the major factor behind the association of ACE genotypes and ESRD patients from north India. However, no significant differences of the renal function parameters (serum creatinin and protein urea) among the DD and non DD genotypes suggests that this variant might not be a factor involved in the causation of renal damage but could have aggravated or related to the progression of the disease. However, being a referral tertiary care centre, most of the patients reported to us were from outside and had incomplete records of various parameters related to progression of the diseases. Hence due to the non-availability of various data points required for the regression analysis of serum creatinin profile, we have not been able to evaluate the role of different ACE genotypes in the progression of the disease as suggested by McLaughlin et al, 1996. [35].

Various reports are available supporting that how the presence of DD genotype operates at cellular level leading to hypertensive state and renal diseases [28]. Caucasians with DD genotype have serum ACE levels and intra-cellular ACE activity twice than those of II genotype [22,36]. High ACE activity leads to increased Angiotensin II levels that promote expression of growth factors and proliferation of mesanglial cells and matrix leading to glomerusclerosis [27]. Incidentally, human genetic variation studies based on autosomal, Y-chromosomal and mt-DNA markers have suggested that north Indians carry high frequency of Caucasian specific mutations and haplotypes [37,38]. Furthermore, the phylogenetic assessments based on the neutral markers have also shown the clustering of north Indians with other Caucasian populations [39,40].

In experimental models of chronic renal disease [41] and in human diabetic nephropathy [42], pharmacological blockade of ACE significantly slows down the rate of decline in renal function. However, the data regarding the relationship between the ACE inhibition and DD genotype has been conflicting. A good correlation was found in IgA nephropathy and diabetic nephropathy [27] but was not confirmed in primary glomerulonephritis [43] and proteinurea [44]. In the present study, we have not been able to deduce the association of ACE inhibition and DD genotype due to non-availability of the information of anti-hypertensive therapy.

In addition to the non availability of multiple values of various renal function parameters and information of the anti-hypertensive therapy, the results of the present study may have also been influenced by the study design and composition of the sample population. Regarding study design, it may be possible that being a single centre study, the samples are over representative of a particular genotype secondly it has been widely accepted that the Indian society is fragmented into numerous sub-groups identified by the name of 'caste' and hence there is a high possibility that the social structuring and stringent marital practices since last 3–4 Ky have also resulted into genetic structuring. We suggest that multi centric studies involving a much higher number of subjects and including controls from different socio-cultural strata will lead to validate the strong association found in the present study.

## Conclusion

Conclusively, ACE gene polymorphism appears to be an important genetic determinant in causation and progression of renal diseases and ACE DD genotype was found to be strongly associated with ESRD among north Indians. Further studies in this regard will open a plethora of options like timing, type and doses of anti-hypertensive therapy. Incorporation of such approaches will allow an advance anticipation of the clinical outcome and can lead to a shift from "One treatment fits all" approach.

## Competing interests

The author(s) declare that they have no competing interests.

## Authors' contributions

GT collected all the samples and has performed experiments. PD has compiled the results and carried out data analysis. FK has interpretated the data and has written the manuscript. RKS and PBV were involved in the patient work up. SA conceptualized the paper and provided important intellectual inputs in the interpretation of the data and preparation of the manuscript. All the authors have read and approved the manuscript.

## Pre-publication history

The pre-publication history for this paper can be accessed here:


